# The INeS study: prevention of multiple pregnancies: a randomised controlled trial comparing IUI COH versus IVF e SET versus MNC IVF in couples with unexplained or mild male subfertility

**DOI:** 10.1186/1472-6874-9-35

**Published:** 2009-12-18

**Authors:** Alexandra J Bensdorp, Els Slappendel, Carolien Koks, Jur Oosterhuis, Annemieke Hoek, Peter Hompes, Frank Broekmans, Harold Verhoeve, Jan Peter de Bruin, Janne Meije van Weert, Maaike Traas, Jacques Maas, Nicole Beckers, Sjoerd Repping, Ben W Mol, Fulco van der Veen, Madelon van Wely

**Affiliations:** 1 Department of Obstetrics and Gynaecology, Academic Medical Centre, University of Amsterdam, Amsterdam, The Netherlands

## Abstract

**Background:**

Multiple pregnancies are high risk pregnancies with higher chances of maternal and neonatal mortality and morbidity. In the past decades the number of multiple pregnancies has increased. This trend is partly due to the fact that women start family planning at an increased age, but also due to the increased use of ART.

Couples with unexplained or mild male subfertility generally receive intrauterine insemination IUI with controlled hormonal stimulation (IUI COH). The cumulative pregnancy rate is 40%, with a 10% multiple pregnancy rate.

This study aims to reveal whether alternative treatments such as IVF elective Single Embryo Transfer (IVF e SET) or Modified Natural Cycle IVF (MNC IVF) can reduce the number of multiple pregnancy rates, but uphold similar pregnancy rates as IUI COH in couples with mild male or unexplained subfertility. Secondly, the aim is to perform a cost effective analyses and assess treatment preference of these couples.

**Methods/Design:**

We plan a multicentre randomised controlled clinical trial in the Netherlands comparing six cycles of intra-uterine insemination with controlled ovarian hyperstimulation or six cycles of Modified Natural Cycle (MNC) IVF or three cycles with IVF-elective Single Embryo Transfer (eSET) plus cryo-cycles within a time frame of 12 months.

Couples with unexplained subfertility or mild male subfertility and a poor prognosis for treatment independent pregnancy will be included. Women with anovulatory cycles, severe endometriosis, double sided tubal pathology or serious endocrine illness will be excluded.

Our primary outcome is the birth of a healthy singleton. Secondary outcomes are multiple pregnancy, treatment costs, and patient experiences in each treatment arm. The analysis will be performed according tot the intention to treat principle. We will test for non-inferiority of the three arms with respect to live birth. As we accept a 12.5% loss in pregnancy rate in one of the two IVF arms to prevent multiple pregnancies, we need 200 couples per arm (600 couples in total).

**Discussion:**

Determining the safest and most cost-effective treatment will ensure optimal chances of pregnancy for subfertile couples with substantially diminished perinatal and maternal complications. Should patients find the most cost-effective treatment acceptable or even preferable, this could imply the need for a world wide shift in the primary treatment.

**Trial registration:**

Current Controlled Trials ISRCTN 52843371

## Background

Since the 1980s the percentage of multiple pregnancies in the Netherlands has increased, as it has in most Western European countries. The tendency of women to delay starting a family until an advanced age with subsequent higher chances of spontaneous multiple pregnancy, is a major cause of this [1]. Also, the increase in assisted-reproduction techniques such as intra-uterine insemination (IUI) and in-vitro fertilisation (IVF) has contributed significantly tot the higher number of multiple pregnancies [2]. Multiple pregnancy is the most frequent and most serious iatrogenic complication of assisted reproductive technologies [3,4].

Women carrying multiple pregnancies have an increased risk of numerous ante- and postpartum complications, such as pregnancy-related diabetes and pre-eclampsia (12%). The chief risk for the infant is preterm birth with an incidence approaching 43%. Prematurely delivered children are prone to suffer from respiratory distress syndrome, bronchopulmonary dysplasia, intraventricular bleeding and pneumonia. Furthermore, the prevalence of growth retardation (42 to 57%) is much higher within children delivered from multiple pregnancies. Altogether, these tribulations lead to high morbidity and a mortality rate of about 3% [5,6]. Admission costs to the neonatal intensive care unit are high and result in a two to four fold increased cost per child born. Additionally, the long-term costs due to handicaps like spastic cerebral palsy are substantial, and subsequently a burden for society.

Both opinion leaders and members of the European and American Societies for Reproductive Medicine (ESHRE and ASRM) consider multiple pregnancies following ART unacceptable for patients and society at large. The presidents of the ESHRE and ASRM have urged their members to prioritize research to prevent multiple pregnancies following assisted reproductive techniques.

Of subfertile couples no less than 50% are diagnosed with unexplained infertility or mild male subfertility, and particularly at risk for multiple pregnancy. In approximately half of these couples the prognosis to conceive spontaneously is unfavourable. In this study we define an unfavourable prognosis as chances of spontaneous conception below 30% in the next 12 months according to the model of Hunault [7]. In couples with a favourable or intermediate prognosis of treatment independent pregnancy, it has been established that expectant management during six months is most opportune [8,9].

However, in couples with an unfavourable diagnosis there is ambiguity about the preferred first line treatment. At present, the recommended fertility treatment in these patients is intra-uterine insemination (IUI) with controlled ovarian hyperstimulation (COH) over six cycles (guideline NVOG). Evidence to support this was established in two systematic reviews where IUI in the natural cycle was less effective than IUI with COH [10,11]. A recent randomised controlled trial found that there was no significant difference in live birth rate, whether stimulation was performed using clomiphene citrate (CC) or gonadotropins [12].

IUI with either form of controlled ovarian stimulation results in considerable pregnancy rates 12.7% (Anderson 2008) at the expense of a large number of multiple pregnancies. National registries in the Netherlands show that multiple pregnancy rate after IUI with or without COH is about 10% [13]. The multiple pregnancy rate after IVF Double Embryo Transfer (DET) is about 25%, and results generated from European registers by ESHRE show multiple delivery rates for IVF are still around 23%. To eradicate this unacceptable consequence of the current ART-practice, every effort has to be put into preventing multiple pregnancies following assisted reproductive techniques.

Since publication of the guideline two alternative strategies have been developed. Firstly, the modified natural cycle IVF (MNC-IVF) was evaluated by the research group from the University Medical Center Groningen. The cumulative clinical pregnancy rate was 37% after six cycles. Live birth rate was 32% per patient. There were 4 multiple pregnancies [14]). Currently MNC is offered as a first line treatment to IVF patients in their clinic.

Also, IVF with elective single embryo transfer (eSET) has been evaluated as a policy to prevent multiple pregnancies. Cumulative pregnancy rates after the embryo transfer of one good quality embryo have been described to be over a 30% [15-17] whereas the multiple pregnancy rates have been low, around 2-3%.

It is clear that multiple pregnancies can largely be prevented by with MNC-IVF and IVF-eSET. However, prevention of multiple pregnancies will only be opportune when high delivery rates can be maintained. Presently, the main problem is that evidence to conclude which one of these treatments is most effective, and cost efficient, is lacking. Also, up to this date, there has been no large randomised controlled trial investigating patient preference comparing these different treatments types. If alternative treatment types are safer and preferable from a patient point of view, this could lead to a shift in standard practice in this population.

## Objective

To study whether MNC IVF and IVF COH can prevent multiple pregnancies, and are effective alternatives to IUI COH in terms of a healthy singleton, multiple pregnancies, costs and patient preferences.

## Methods

### Participating centres

This study is a multicentre randomised controlled trial in IVF centres and their affiliated clinics in The Netherlands. Inclusion started in January 2009.

### Inclusion criteria

Couples with female age between 18 and 38 years, diagnosed with unexplained or mild male subfertility, failure to conceive within at least 12 months of unprotected intercourse, and poor prognosis, are eligible. In this study, a poor prognosis is defined as a chance of spontaneous pregnancy below 30% within 12 months as calculated by the validated model of Hunault [18,19], or failure to conceive within at least 3 years of unprotected intercourse. Mild male subfertility is defined as pre-wash total motile sperm count above 10 million or a post-wash total motile sperm count above 1 million.

Patients with polycystic ovary syndrome or any other anovulation, double-sided tubal pathology, endocrinopathological disease (Cushing syndrome, adrenal hyperplasia, hyperprolactinemia, acromegaly, imminent ovarian failure, premature ovarian failure, hypothalamic amenorrhea, diabetes mellitus (type I) will not be included.

### Ethical considerations

Approval for this study was obtained from the Medical Ethical Committee of the Academic Medical Centre and from the Central Committee on Research involving Human Subjects (CCMO), The Netherlands. In patients fulfilling the inclusion criteria, written informed consent is obtained before randomisation is carried out. Women refusing participation are registered.

### Randomisation

Randomisation is performed by accessing a central internet-based randomisation program and is stratified for hospital.

### Interventions

**Figure 1:**
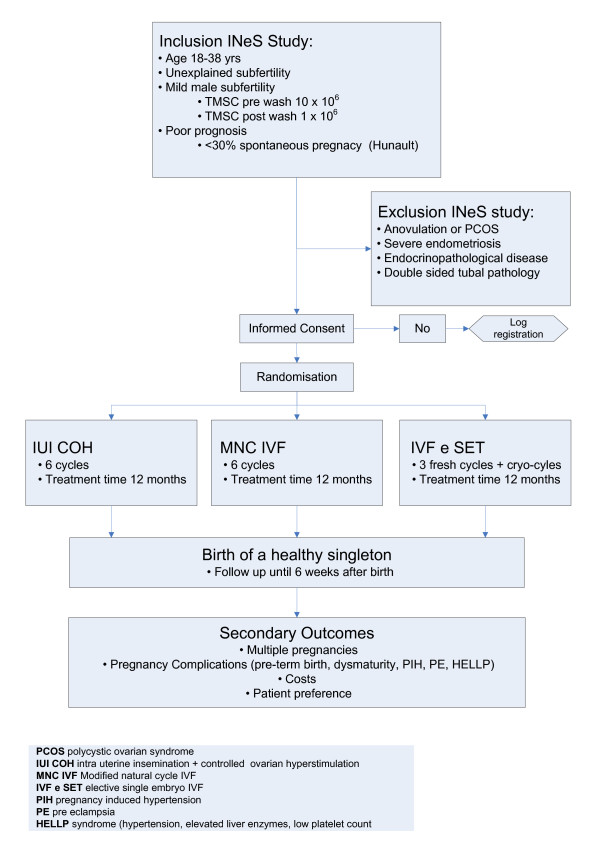
**Flowchart of study**.

Couples are allocated to a treatment strategy consisting of 6 consecutive cycles of IUI-COH or 6 cycles of MNC-IVF or 3 cycles of IVF-eSET plus subsequent cryo-cycles. Couples are treated until pregnancy occurs within a treatment time horizon of 12 months. (Figure 1).

Prior to start of IUI-COH patients will undergo Chlamydia antibody testing, hysterosalpingography and/or laparoscopy to exclude double sided tubal occlusion.

In IUI-COH the controlled ovarian hyperstimulation can be performed with 100 mg clomiphene citrate or by daily subcutaneous injections of 75 IU FSH starting from cycle day three or four onward. The follicular growth is strictly monitored by transvaginal ultrasound. When at least one follicle with a diameter of 17 or 18 mm is present, final oocyte maturation is induced by the administration of 5.000 IU human chorionic gonadotropin (hCG) (Pregnyl, Organon, Oss, The Netherlands), and 36 hours thereafter IUI will be performed. HCG administration and IUI will be withheld when monitoring reveals the growth of more than three follicles with a diameter of 16 mm, or more than five follicles with a diameter of 12 mm. Semen samples are processed within one hour after ejaculation, using a density gradient centrifugation followed by washing with culture medium and is subsequently used for insemination.

In MNC-IVF the oocyte that develops spontaneously is used for IVF. The cycle is minimally modified with a GnRH antagonist to prevent untimely ovulations, together with FSH to prevent collapse of the follicle and a concomitant fall in estradiol levels. Ultrasound monitoring will be started on cycle day 8 or 10 and repeated daily or every other day, according to the size of the lead follicle. Follicle diameter will be measured in three perpendicular planes, and the mean value will be taken. When a lead follicle with a mean diameter of at least 14 mm is observed, daily injections of 0.25 mg of a GnRH-antagonist together with 150 IU FSH are started. GnRH-antagonist will be continued up to and including the day of ovulation triggering. FSH will be continued up to the day of ovulation triggering. Patients will be instructed to have their injections at the same time daily, to ensure a 24 hour interval between injections. Ovulation triggering will be achieved by subcutaneous injection of 10 000 IU of hCG (Pregnyl^®^, Organon, Oss, the Netherlands) when a follicle with a diameter of 17 to 18 mm is observed.

Blood samples will be taken for assessment of serum concentrations of LH and E2 on the days that ultrasound was performed. Cycles where an LH-rise of > 30.0 IU/L is noticed at a follicle size of < 15 mm will be cancelled. In cases where an LH-rise of > 30.0 IU/L is noticed, HCG is given and follicle aspiration will be performed the next day. There will be no flushing of the follicle. Only large (dominant) follicles will be aspirated. Embryo transfer will be performed on the second or third day after oocyte retrieval. Usually one oocyte is obtained, and one embryo is placed in utero. Should there be more than one good quality embryo, the surplus embryos will be cryo preserved.

For luteal support, HCG 1500 IU (Pregnyl^®^, Organon, Oss, the Netherlands) will be given five, eight and eleven days after oocyte retrieval.

For IVF-eSET women will undergo controlled ovarian hyperstimulation after down-regulation with a GnRH agonist in a long protocol with a midluteal start or with a fixed start antagonist protocol starting on day two. Controlled ovarian hyperstimulation is started with 150 IU FSH. Treatment is continued until at least 2 follicles > 18 mm have developed. Ovulation is induced by 10.000 IU human chorionic gonadotropin hormone (hCG) (Pregnyl^®^, Organon, Oss, The Netherlands) and cumulus-oocyte complexes will be recovered by transvaginal ultrasound-guided retrieval 36 hours thereafter. Embryos will be scored with the use of validated morphological scoring criteria at the time of fertilization (pronuclear morphology) and daily until the time of transfer. Embryos will be assessed for their morphology daily by an embryologist/IVF-technician using an Olympus IX71 inverted microscope or another equivalent microscope equipped with Relief Contrast optics at a magnification of 320 x. On day two, three or four, one embryo will be selected for transfer if one or more embryos of good quality are available. If no good quality embryos are available two embryos will be transferred. Non-transferred good quality embryos will be cryo-preserved. Embryo scoring will be done according to the participating laboratories' own protocol. When implantation is not successful or early miscarriage occurs the frozen embryos are thawed and transferred. Again, only one embryo will be transferred per frozen-thaw cycle if it is of good quality.

Before starting a new treatment cycle with IUI-COH, MNC-IVF or IVF-eSET, women will undergo a pregnancy test (hCG measurement in serum or urine). If no pregnancy has manifested the next treatment cycle is started according to protocol. In case of a positive pregnancy test women will be monitored using ultrasound visualisation during their pregnancy. Monitoring will take place at five to nine weeks of amenorrhea to check whether an intrauterine gestational sac is present, i.e. a clinical pregnancy. Subsequently monitoring will take place at 11 to 12 weeks amenorrhea to register the presence of an intrauterine gestational sac with foetal heart beat, i.e. an ongoing pregnancy.

### Follow up

#### Short term follow up

As the primary outcome is birth of a healthy child resulting from a pregnancy that was established in the first 12 months after randomization, each child will be assessed at 6 weeks after the expected day of delivery. For example, a child that is born at a gestational age of 38 weeks will be assessed at 8 weeks after birth, whereas a child born after a gestational age of 30 weeks will be assessed 16 weeks after birth. Parents will be contacted by telephone to enquire on the delivery and the health of the child. When necessary, child health centres and or paediatricians will then be contacted for specific information. All children with severe morbidity will be evaluated and classified by a panel of neonatologists. Detailed information on maternal complication will be obtained from the obstetrician treating the woman concerned.

Certainly not all couples will complete the 12 months of treatment. Drop-outs will largely represent normal patient flow. We aim to keep track of all drop-outs and to document the reason for the drop-out in the database.

#### Long term follow up

We will not be able to follow the children during their lives within the context of this study. However, the health status and subsequent development of children born after IVF and ICSI seems to be comparable to those after spontaneous conception up to the age of 5 years [20]. Furthermore it was recently shown that couples in whom eSET was applied, did not have an unfavourable outcome of their singleton baby when compared to spontaneous singletons [21].

### Outcome measures

#### Primary outcome measure

The primary outcome is the birth of a healthy singleton. A child will be considered healthy when born at term with a normal birth weight, without congenital anomalies [22] and with a normal paediatric examination and development assessment at 6 weeks of age.

#### Secondary outcome measures

Naturally, multiple pregnancy, defined as registered heartbeat of at least two foetuses at 12 weeks of gestation is a secondary outcome. A multiple birth in which al least one of the children has congenital anomalies, will considered as failure of the strategy.

Further secondary outcomes are clinical pregnancy, defined as any registered embryonic heartbeat at sonography; neonatal mortality; pregnancy complications (preterm birth < 37 weeks, birth weight < 2.500 gram, Pregnancy Induced Hypertension (PIH), (pre-) eclampsia, HELLP) costs and patients' preferences.

Patients' preferences are assessed by an (online) questionnaire using a discrete choice experiment (DCE) based on characteristics of both interventions and will be compared with a control group, recruited among women visiting the infertility clinics of the participating hospitals.

### Background and Demographic Characteristics

To assess whether the treatment groups were balanced, the study populations will be compared for baseline measurements including female age, type of infertility (primary/secondary), duration of infertility, intoxications, body mass index, as well as sperm analysis according to WHO standards.

### Analysis

The analysis of all outcomes is on an intention to treat basis. The analysis will be a cost-minimisation when the effectiveness is comparable in all groups. In case of differences in effectiveness a cost-effectiveness study will be performed. Couples will be treated within a time horizon of 12 months

Differences in birth rate per group will be expressed as relative risks with corresponding 95% confidence intervals. A formal test of the difference in pregnancy rate will be performed using chi-square test statistics. Birth rates over time will be compared using life tables.

The medical, economical and social dilemma concerns children with handicaps, which are mostly due to pre-term birth. A literature search will therefore be performed to estimate live-long costs due to handicaps. We will subsequently perform a scenario analysis, in which we will model the costs and effects of the three strategies in this perspective.

### Economic Evaluation

We plan an economic evaluation alongside the clinical trial. A distinction will be made between costs of medical interventions (direct costs) and costs resulting from productivity losses (indirect or time costs). Standardised unit costs will be calculated for all centres based on actual expenses made during the study. Subsequently, unit costs will be applied to resource use as observed in the participating centres. Resource utilisation will be documented using individual patient data in the case record forms. In addition, each woman will receive questionnaires at 4 weeks, 8 weeks, 12 weeks, 24 weeks, 36 weeks and 48 weeks after treatment start for details on associated direct costs of professional care, and on indirect costs like transportation and productivity loss. Resource unit prices will reflect the unit of staff, materials, equipment, housing, depreciation, and overhead. Productivity loss will be valued using Dutch reference data (handbook of the Dutch health Council). Costs will be presented in Euros.

As the neonatal morbidity and mortality is likely to depend on the number of multiple births, we will perform sensitivity analyses in which we will vary the number of multiple pregnancies in the IUI-COH strategy. Furthermore, sensitivity analyses will be performed on costs, pregnancy rates of the different treatment strategies and the valuation of different outcomes (no child, handicapped child, healthy child).

### Power calculation

Considering an expected birth rate of a healthy child after 12 months of treatment of 40% in all three treatment groups, with an alpha of 5% and a beta of 20%, 190 patients per group are required to exclude a difference of 12.5% or more to the detriment of MNC-IVF and IVF-eSET. To account for an estimated early drop-out of 5% we will include 200 patients per group.

## Discussion

Each year about 5000 couples in the Netherlands are diagnosed with unexplained or mild male and an unfavourable prognosis [23]. As stated earlier, in these couples there is ambiguity about the preferred first line treatment.

The incidence of multiple pregnancies with the standard treatment is substantial. This has far reaching consequences regarding maternal and neonatal morbidity, but also with regard to societal costs. Therefore alternative treatments need to be considered. To this date no large randomised controlled trials which have compared IUI COH with IVF e SET, and MNC IVF in couples with unexplained or mild male subfertility in order to prevent multiple pregnancies.

## Competing interests

The authors declare that they have no competing interests.

## Authors' contributions

AJB is responsible for the overall logistical aspects of the trial and drafted the paper. FvdV, MW, and BMW designed the trial, were responsible for the development of the protocol, applied for a grant and have overall responsibility for the trial. All authors read and approved the final paper.

## Pre-publication history

The pre-publication history for this paper can be accessed here:

http://www.biomedcentral.com/1472-6874/9/35/prepub
